# Sugary drinks taxation, projected consumption and fiscal revenues in Colombia: Evidence from a QUAIDS model

**DOI:** 10.1371/journal.pone.0189026

**Published:** 2017-12-20

**Authors:** Juan Carlos Caro, Shu Wen Ng, Ricardo Bonilla, Jorge Tovar, Barry M. Popkin

**Affiliations:** 1 Dept. of Health Policy and Management, Gillings School of Global Public Health, University of North Carolina, Chapel Hill, North Carolina, United States of America; 2 Carolina Population Center and Dept. of Nutrition, Gillings School of Global Public Health, University of North Carolina, Chapel Hill, North Carolina, United States of America; 3 Centro de Investigaciones para el Desarrollo, Universidad Nacional, Bogotá, Colombia; 4 Facultad de Economía-CEDE, Universidad de Los Andes, Bogotá, Colombia; SOAS, University of London, UNITED KINGDOM

## Abstract

The global shift towards diets high in sugar-sweetened beverages (SSBs) is linked to higher prevalence of obesity, diabetes and most other non-communicable diseases. In Colombia, one out of every two people was overweight or obese by 2010. This study estimates price-elasticities from a Quadratic Almost Ideal Demand System model, using the 2006–2007 Colombian Income and Expenditure survey. The food groups that were jointly considered were: unsweetened unflavored milks; coffee and tea; sugar sweetened beverages (SSBs); sweets and candies (including sugar); dairy products; meats and animal-based products; grains based staples; fruits and vegetables; and condiments and snacks. We take into account the high proportion of households not purchasing specific food and beverage groups (censored data) and endogeneity on both prices (as unit values) and total expenditure. Unhealthy beverages are price-elastic (-1.61 for SSBs) meaning that the change in consumption is proportionally larger with respect to a change in price. Also, there is a high complementarity among SSBs and major food groups (grains, meats and fruits and vegetables). In Colombia, the design of a meaningful tax to influence healthier diets is a next critical step. This study also shows that a tax of 20% on SSBs should prove to be effective, and can yield revenues of about 1% of the Colombian government’s total annual fiscal revenue, which can potentially be directed towards public health promotion and investments.

## Introduction

There is a growing concern in developing countries regarding how economic transitions influence the prevalence of obesity and other non-communicable diseases (NCDs) [1–5]. Obesity and overweight prevalence in Colombia has increased dramatically in the recent years. The 2010 National Nutritional Status Survey estimated that the prevalence of overweight and obesity is 51.1% among adults and 17.5% among children 5-17y [6–8].

It is clear from a large body of epidemiological research that SSBs represent a major cause of the health problems that include obesity, diabetes and dental caries [9–11]. These findings are consistent with the global consensus that links SSBs and energy dense ultra-processed foods as leading risk factors associated with obesity and overweight [12, 13]. Furthermore, in Colombia and elsewhere, sugar sweetened beverages (SSBs) are being fed to infants in lieu of both breast-milk and healthier weaning [14–17].

Several policies have been recommended and/or implemented to reduce SSBs consumption in different countries and contexts, mainly focusing on front-of-package (FOP) food profiling and labelling, marketing restrictions, taxation, and removal of these beverages from public institutions [18–23]. Mexico, France, 13 of the Western Pacific Islands, Hungary, and Denmark are among countries that have recently passed taxation laws to reduce consumption of these beverages [24–28]. A recent report from the World Health Organization that pulled together evidence from 11 systematic reviews support the use of fiscal policies such as taxing sugary drinks including SSBs [29].

Until the evaluations of the first few countries to implement such taxes, such as in Mexico [30], Hungary [26] and Denmark [31], there had been limited evidence of how taxing SSBs would affect consumption except from model-based simulations. In the case of Colombia, even model-based estimates on the potential of SSB taxes do not exist. In this context, price-elasticities estimated from demand system models are a key element to measure the potential impact of fiscal policies on expenditure for specific food groups. Although other factors such as households’ out-of-model behavior and industry marketing response also need to be considered when assessing counterfactuals for policy simulation, price-elasticities constitute a useful benchmark to analyze fiscal policy outcomes.

This study jointly estimates own- and cross- price-elasticities of SSBs and unhealthy foods for Colombian urban households using the most recent nationally representative income and expenditures survey from 2006–2007, and projects the simulated effects of SSB taxation on consumption and tax revenues. Our paper adds on the existing literature regarding quadratic almost ideal demand system models (QUAIDS), its applications to SSBs taxation in Colombia, and what it means in terms of potential fiscal revenue. Results of this study contribute to the discussion on the current SSB tax framework and how it may reduce SSB consumption-, and hypertension, in Colombia.

## Materials and methods

### Data

For model estimation and price elasticity calculations we used the National Income and Expenditure Survey (ENIG, Spanish acronym) collected between 2006–2007, by the National Administrative Department of Statistics [32]. The ENIG is a cross-sectional survey that contains household level information regarding quantities and expenditures on all items used to construct the Consumer Price Index weights, and also reports socioeconomic and demographic information (used to define poverty lines, among other applications). The ENIG has a probabilistic, stratified, two stage sample design. The sample size from the raw data is 35,998 households. The survey is representative at the department level for 24 different metro areas covering 226 municipalities. Limiting the sample to households with complete sociodemographic and food purchase data, resulted in a sample size of 33,824 households (94% of original sample) in this analysis.

In order to project SSB consumption trends and potential tax revenues derived from the implementation of an SSB tax, additional sales data from Euromonitor International was used, including on-trade (final consumer) and off-trade (food service such as restaurants) historical and forecasted sales for drinks with added sugar [33]. Also, to provide context for the tax revenue, official fiscal revenue information was used to forecast total revenue each year, until 2020 [34].

### Variable construction

For this study, nine mutually exclusive groups of food and beverages were defined, in order to observe potential complementarities and substitutions: (1) unsweetened and unflavored milk; (2) coffee, bottled water and tea; (3) SSBs (concentrated and ready-to-drink); (4) sweets and candies (including traditional sweets, raw and refined sugar); (5) dairy products (cream, yogurt, etc.); (6) grains and flour-based products; (7) meats and animal-based products, including seafood; (8) fruits and vegetables; and (9) condiments and snacks (this group included all other foods, commonly referred as residual good).

Food expenditure shares for each group were calculated by summing the expenditures within each group and then dividing it by total expenditure of the nine categories. Unit values of prices were calculated as the ratio of expenditure over quantity for each group, and then aggregated to obtain estimates at the municipal level (dataset included 226 municipalities). Further adjustments were made for items that are consumed in a reconstituted form from powder or concentrate [35]. To account for household composition, adult equivalent units (AE) calculations were done as follows: for children under 5 years old equals to 0.77 AE; children from 6 through 12 years old equals to 0.80 AE, and adolescents from 13 to 18 years old accounts for 0.88 AE. This is standardized approach refined by the United Nations Food and Agriculture Organization and the World Health Organization [36].

### Quadratic Almost Ideal Demand System (QUAIDS)

We seek to understand consumer sensitivity to price changes across different socioeconomic status (SES) subpopulations. Thus, it is relevant to consider a utility-based structural model, which models individual behavior in response to prices, specifically uncompensated demand (which maximizes utility given prices and income or wealth). To do this, we estimate the quadratic extension of the Almost Ideal Demand System model [37], introduced by [38], QUAIDS for short, which allows more flexibility over the income-expenditure (Engel) curves. The goal of demand analysis is to model households’ expenditure patterns on a group of related items in order to obtain estimates of price elasticities. This is the standard approach used by economists to study SSB, tobacco, alcohol and other types of price effects on consumer purchasing behavior. It models the direct own price effects on SSB purchases while also considering the direct price elasticity of other beverages and/or foods and the cross-price elasticities among all the products in the system [30, 39–43]. We assumed weak budget separability, therefore modelling the total demand for food at home, divided in nine food groups. The model in its budget share form is defined as follows:

$$\begin{array}{c} w_{i}=\alpha_{i}+\sum_{j\in I}\gamma_{ij}\ln p_{j}+\beta_{i}ln(\frac{m}{a(p)})+\frac{\lambda_{i}}{b(p)}{\left(\ln\left(\frac{m}{a(p)}\right)\right)}^{2}\;\forall \;i\in I \end{array}$$ (1)

$$\begin{array}{c} \alpha_{i}=\alpha_{0i}+\sum_{k\in K}\rho_{ik}z_{k} \end{array}$$ (2)

$$\begin{array}{c} \ln a(p)=\alpha_{0}+\sum_{j\in I}\alpha_{j}\ln p_{j}+\frac{1}{2}\sum_{l\in I}\sum_{j\in I}\gamma_{lj}\ln p_{l}\ln p_{j} \end{array}$$ (3)

$$\begin{array}{c} b(p)=\prod_{j\in I}p_{j}^{\beta_{j}} \end{array}$$ (4)

Where *w*_*i*_ and *p*_*i*_ are the budget share and price of the food item group *i*, and *m* is the total food expenditure per household. *I* represents the set of all food groups. *z*_*k*_ is a set of sociodemographic variables introduced to allow household heterogeneity. As noted by [37], linear constraints over parameters are required for the model to be consistent with economic demand theory. Specifically, homogeneity of degree zero on prices (if prices and income change in the same ratio, demanded quantities are unaffected) and symmetry (substitution or complementary effects between goods are symmetrical in direction and magnitude). Also, the demand system should add-up to one, since it is expressed in budget form, i.e. ∑_*i*∈*I*_ *w*_*i*_ = 1. Therefore, the constraints over parameters that should be imposed are the following:

$$\begin{array}{c} \sum_{i\in I}\alpha_{i}=1,\;\sum_{i\in I}\beta_{i}=0,\;\sum_{i\in I}\lambda_{i}=0,\;\sum_{i\in I}\gamma_{ij}=0\;\forall \;i\in I \end{array}$$ (5)

$$\begin{array}{c} \sum_{j\in I}\gamma_{ij}=0\;\forall \;i\in I,\;\gamma_{ij}=\gamma_{ji}\;\forall \;i,j\in I \end{array}$$ (6)

$$\begin{array}{c} \sum_{i\in I}\rho_{ik}=0\;\forall \;k\in K \end{array}$$ (7)

### Endogeneity and censoring approach

The model used for this analysis considers two very important issues in the demand system estimation, namely the non-negligible proportion of households that do not report purchases on many of the categories of foods and beverages we consider (i.e., censoring), and the endogeneity of both prices derived from unit values and total expenditure. Endogeneity of total expenditure is likely since shares of consumption and total expenditure are determined jointly. Also, since National Income and Expenditure survey does not contain price information, we use the ratio of expenditure to quantity for each item (unit values), which may introduce endogeneity, household level heterogeneity, and measurement error [44].

To account for potential price endogeneity, we follow the approach described in [45], assuming that households in the same geographic area (municipalities in this case) face the same prices. In particular we can write the equation for unit values (*v*_*ihc*_) as follows:

$$\begin{array}{c} {\ln\;v}_{ihc}=\ln\;p_{ihc}+\epsilon_{i}\;\ln\;q_{ihc}+\sum_{l\in L}\eta_{il}z_{lhc}+u_{ihc} \end{array}$$ (8)

Where *h* indexes households, distributed across *c* municipalities. Then, imposing the assumption *p*_*ihc*_ = *p*_*ic*_ is possible to estimate the equation above demeaning values at the municipal cluster level. As demographic controls we used age, gender, and education of the head of household, and household size, as well as dummy variables indicating the presence of children and single households. After obtaining the estimates, we obtain price estimates at the municipal cluster level, such that:

$$\begin{array}{c} {\ln\hat{p}}_{ic}=\ln v_{ic}-\hat{\epsilon_{i}}\ln q_{ic}-\sum_{l\in L}\hat{\eta_{il}}z_{lc} \end{array}$$ (9)

To address potential total expenditure endogeneity, we estimated an ordinary least squares regression for total expenditure over a set of explanatory variables, including socio-economic status (SES) and geographic region of the household, as well as an index for access to basic amenities (gas, electricity and drinking water), age, gender, and education of the head of household, and household size. Afterwards, we used the residuals of that model as an additional explanatory variable in the demand system, as proposed by [38].

Household budget and expenditure surveys can include zero expenditure in certain food groups due to a number of reasons such as non-availability, optimal choice (i.e. corner solutions to the household optimization problem), or infrequent purchases, not captured within the time frame of the survey. Because these reported zeroes are likely selective, we can formalize the underlying model for consumption choice as follows:

$$\begin{array}{c} D_{i}=1(\sum_{j\in I}\tau_{j}\ln p_{j}+\pi_{i}m+\sum_{k\in K}\theta_{k}z_{k}>v_{i}) \end{array}$$ (10)

$$\begin{array}{c} w_{i}=D_{i}w_{i}^{*} \end{array}$$ (11)

Where *D* is an indicator function for the decision of consumption, w* represents the unobserved latent demand, and *v* is an error term. In practice, several approaches to solve the censoring problem have been developed. In particular [46] propose a two-step process to estimate the model. First, a discrete model for the consumption choice is estimated and used to predict the cumulative distribution (Φ) and probability density functions (*ϕ*) for each household. Then, this information is used in the second step to modify the demand system defined in Eq 1 into:

$$\begin{array}{c} w_{i}^{*}={\hat{\Phi }}_{i}w_{i}+\delta_{i}\hat{\phi_{i}}+u_{i} \end{array}$$ (12)

Where *δ*_*i*_ are parameters to be estimated, and *u*_*i*_ is the error term. The system described in Eq (12) is often referred as the augmented QUAIDS model.

### Price elasticities

In all cases, after estimating the parameters, the uncompensated own and cross price-elasticities were computed for the mean values of the variables. Elasticities represent the sensitiveness of expenditure due to price changes, where an absolute value of one implies that both vary in the same proportion (in percent terms). Values lower (greater) than one, implies that expenditure shifts less (more) in proportion to a change in price. Given the two-step censoring correction, we adjust the elasticity formula for the base model, following the inclusion of both direct effects (conditional on positive demand) and indirect effects or the intensive margin (change in likelihood to consume), as noted by [47] and [48]. Therefore the price elasticity can be estimated from the following equation derived in [49]:

$$\begin{array}{c} e_{ij}=-\delta_{ij}+\frac{1}{w_{i}^{*}}[\Phi_{i}(\gamma_{ij}-(\beta_{i}+\frac{2\lambda_{i}}{b(p)}\ln\left(\frac{m}{a(p)}\right))(\alpha_{j}+\sum_{k}\gamma_{jk}ln\;p_{k})-\frac{\lambda_{i}\beta_{j}}{b(p)}{\left(\ln\left(\frac{m}{a(p)}\right)\right)}^{2})+\tau_{j}\phi_{i}(w_{i}-\delta_{i}D_{i}^{*})] \end{array}$$ (13)

Where *δ*_*ij*_ is the Kronecker delta (equal to one only for own price elasticities, and zero otherwise) and $D_{i}^{*}$ is the argument of the indicator function in Eq (10).

### Estimation strategy

As discussed in [50] and [51], models like the augmented QUAIDS described in Eq (12) does not longer satisfy the additivity constrains, therefore estimation will be non-invariant to the excluded equation if such restrictions are imposed. If we interpret the error term in Eq (12) as deviations from optimal planning, then we no longer need to assume that $\sum_{i\in I}w_{i}^{*}=1$, and therefore we estimated the system fully. However, since conditional on positive consumption, the budget constrain is bounding, the conditions over the parameters are still imposed to ensure ∑_*i*∈*I*_ *w*_*i*_ = 1 (Eqs (5) and (7)). The estimation process is divided in two stages. First, we estimated individual probit equations for each food group, and then estimated the cumulative and density functions. We used as demographic variables the demographic shifters included in the demand system, excluding age of head of household, to allow identification. In the second stage, we used the predicted variables in the first stage to estimate the model in Eq (12) as Seemingly Unrelated Regression system. We fixed *α*_0*i*_ = 5 < min(ln(*m*)), following the criteria suggested by [38]. Finally, we calculate the price elasticities following Eq (13). In sum, we estimated the QUAIDS two-step censored model consistent with economic demand theory, correcting for endogeneity in prices and total expenditure, defined hereon as our censored model. We used demographic shifters to allow for household heterogeneity; age, education of the head of household, and household size. We conducted robustness checks using an uncensored model with the same specification. We also stratified the analysis using the official SES classification that the Colombian government uses to rank households receiving subsidies (Departamento Administrativo Nacional de Estadistica, 2009). Thus, the full sample was split into two sub-samples: low SES households, and middle-high SES households. This allows us to estimate the potential heterogeneous response to prices across SES groups in Colombia.

All models were estimated by Feasible Generalized Non-linear Least Squares (FGNLS), and standard errors were computed by non-parametric bootstrap with 500 repetitions, using Stata v.14.1 [52].

### Consumption and tax revenue projections

Estimations of projected consumption and tax revenue for 2017–2020 are calculated simulating an ad-valorem (percent according to value) tax on SSBs being implemented from 2017 onwards. We used Euromonitor forecasted yearly gross volume sales data for the 2016–2020 period, and prices adjusted by a flat 3% inflation rate and fixed exchange rate of $3,000 Colombian pesos (COP) to $1 U.S. dollar (USD). Tax revenue for each year is computed as the forecasted volume of sales (after the tax) times the tax rate, using an average price per liter, before the value added tax (currently 16%). We express the results in nominal terms (US$ millions of each year) and as percentage of total expected fiscal revenue each year (based on historical growth trends).

## Results

Table 1 shows socio-demographic descriptive statistics. On average, low SES households are more likely to have male as head of household, younger, and with fewer years of education. Also, low SES households are larger (based on adult equivalents).

**Table 1:** ENIG household demographics (33,824 households).

	Low SES	Mid-high SES	Total Sample
Number of households (% of total)	20562	13262	
Average expenditures (200 USD, 2100 COP = 1 USD)	109.14	133.48	113.32
Gender of household head (0 = female)	0.64	0.62	0.63
Age of household head (years)	46.4	50.2	47.8
Education of household head (years)	10.6	14.7	12.2
Household size (equivalent adults)	4.04	3.47	3.83

Source: Colombian Income and Expenditure Survey (ENIG) 2006–2007. Weighted values. Income groups based on official SES classification as described in the data section (low = 1; mid-high = 2,3,4,5,6).

Table 2 reports percentage of households reporting consumption in each category, the median expenditure per food group and mean unit values across the different SES strata. The SSBs and tea, water and coffee groups have the lowest percentage of households reporting purchases. In contrast, more than 90% of households report buying dairy products, grain based staples and fruits and vegetables. Animal based products, as well as condiments and snacks are the groups with higher mean unit value per kilo within the foods, while SSBs and milk are more expensive than other beverages on average.

**Table 2:** ENIG consumption descriptive statistics (33,284 households).

	Low SES (20,562 obs.)	Mid-high SES (13,262 obs.)	Full sample
Item	Households reporting expenditure >0		
Milk	73.9%	80.6%	76.4%
Tea, water and coffee	61.8%	65.2%	63.0%
SSBs	31.0%	34.9%	32.4%
Sweets and candies	79.1%	72.7%	76.5%
Dairy products (excl. milk)	93.1%	90.2%	91.9%
Grain based staples	95.3%	93.2%	94.4%
Meats and animal-based products	89.4%	85.1%	87.6%
Fruits and vegetables	93.5%	89.2%	91.8%
Condiments and snacks	79.3%	79.1%	79.2%
	Mean expenditure shares		
Milk	8.6%	10.7%	9.5%
Tea, water and coffee	2.6%	2.9%	2.7%
SSBs	2.0%	3.2%	2.5%
Sweets and candies	5.1%	3.8%	4.5%
Dairy products (excl. milk)	13.2%	13.1%	13.2%
Grain based staples	19.1%	16.3%	17.9%
Meats and animal-based products	26.6%	26.7%	26.6%
Fruits and vegetables	19.1%	19.1%	19.1%
Condiments and snacks	3.7%	4.4%	4.0%
	Mean unit values per kilogram or liter (2007 USD, 2100 COP = 1 USD)		
Milk	0.67	0.71	0.69
Tea, water and coffee	0.12	0.13	0.13
SSBs	0.69	0.73	0.71
Sweets and candies	0.94	0.94	0.94
Dairy products (excl. milk)	2.23	2.44	2.32
Grain based staples	1.32	1.52	1.40
Meats and animal-based products	3.52	3.84	3.65
Fruits and vegetables	0.86	0.93	0.89
Condiments and snacks	5.58	5.54	5.56

Source: Colombian Income and Expenditure Survey (ENIG) 2006–2007. Weighted values. SSB: sugar-sweetened beverages. COP: Colombian peso. USD: US Dollar. Income groups based on official SES classification as described in the data section (low = 1; mid-high = 2,3,4,5,6).

In Table 3 we show the mean uncompensated price-elasticities from the censored model. The values along the diagonal reflect own-price elasticities, while the values on the off-diagonal reflect cross-price elasticities. Most food groups have inelastic own-price elasticities (negative and absolute value is smaller than one), but the beverage groups have elastic own-price elasticities (absolute value greater than one). The own-price elasticity of SSBs ready to drink is elastic (-1.62), which means that for a 10% price increase, purchases is estimated to fall by 16%. Other unhealthy items, such as sweets and desserts, show smaller price-elasticities (in absolute value), but these are also one of the groups that account for the lowest proportion of households reporting expenditures and smaller expenditure shares overall. Cross price-elasticities show substitution and complementarity patterns among food groups. In particular, we observe substitutions between SSBs and condiments and snacks, as well as to grains, dairy-based products and fruits and vegetables (positive cross-price elasticities). This means that a price increase on SSBs will have a positive effect on consumption for these groups. We also observe complementarities between SSBs and milk, as well as with sweets and candies (negative cross-price elasticities). On the other hand, we observe similar substitutions for SSBs in relation to the change in price of dairy products, grains and condiments, while also SSB consumption seems to be complementary to meat products’ prices.

**Table 3:** Uncompensated elasticities from QUAIDS censored model (33,824 households).

	Change in price								
Change in quantity	**Milk**	**Tea and coffee**	**SSBs**	**Sweets and candies**	**Diary-based products**	**Grain based staples**	**Meat and animal products**	**Fruits and vegetables**	**Condiments and snacks**
**Milk**	-1.048***	0.116*	-0.162***	-0.127***	-0.052	0.303***	1.085***	0.575***	0.035
	**0.096**	0.061	0.062	0.043	0.06	0.065	0.167	0.15	0.042
**Tea, water and coffee**	0.043	-1.346***	0.074	0.06	0.067	-0.152	-0.7	-0.36	-0.058
	0.143	**0.18**	0.108	0.113	0.103	0.129	0.536	0.498	0.058
**SSBs**	0.018	-0.003	-1.616***	-0.135	0.345***	0.231***	-0.601**	-0.216	0.101**
	0.122	0.064	**0.104**	0.085	0.075	0.086	0.27	0.266	0.041
**Sweets and candies**	-0.088	-0.036	-0.239***	-0.801***	0.212***	0.044	0.554***	0.85***	-0.074**
	0.077	0.051	0.054	**0.068**	0.054	0.056	0.133	0.126	0.036
**Diary-based products**	-0.164***	0.061**	0.12***	-0.059*	-0.94***	0.048	0.321***	0.299***	0.006***
	0.042	0.03	0.033	0.027	**0.038**	0.036	0.105	0.097	0.018
**Grain based staples**	0.042	0.056*	0.058**	-0.064***	-0.01	-0.852***	0.053	0.215**	0.024
	0.037	0.029	0.029	0.025	0.028	**0.032**	0.127	0.11	0.015
**Meat and animal products**	0.081***	0.021	-0.017	-0.027	-0.029	-0.119***	-0.84***	-0.194*	-0.001
	0.024	0.018	0.02	0.024	0.02	0.022	**0.093**	0.081	0.008
**Fruits and vegetables**	-0.111***	0.036*	0.079***	0.026	-0.026	-0.043	-0.347***	-0.961***	0.006
** **	0.032	0.02	0.025	0.024	0.027	0.027	0.094	**0.098**	0.012
**Condiments and snacks**	0.427**	-0.382**	0.361**	0.326***	-0.185	-0.275*	-2.039***	-1.806***	-1.01***
** **	0.19	0.161	0.151	0.1	0.154	0.163	0.426	0.419	**0.123**

Source: Colombian Income and Expenditure Survey (ENIG) 2006–2007. SSB: sugar-sweetened beverages. Bold denote own price elasticities. p<0.1*, p<0.05**, p<0.01***.

Our results show that there are important differences in magnitude and sign of the estimated elasticities when accounting for censoring, reflecting two effects; first, the parameter estimates change due to the specification of the demand system, as noted above, but also the elasticity formula in the censored case explicitly includes the contribution of households with zero purchases (elasticity estimates from the uncensored model are shown in S1 Table). Overall, the results from the censored model are more precise and statistically significant. In particular, the SSBs elasticity is larger in the censored model, indicating that households are quite likely to switch on and off consumption due to changes in price, in addition to change the volume of purchases.

**Fig 1:**
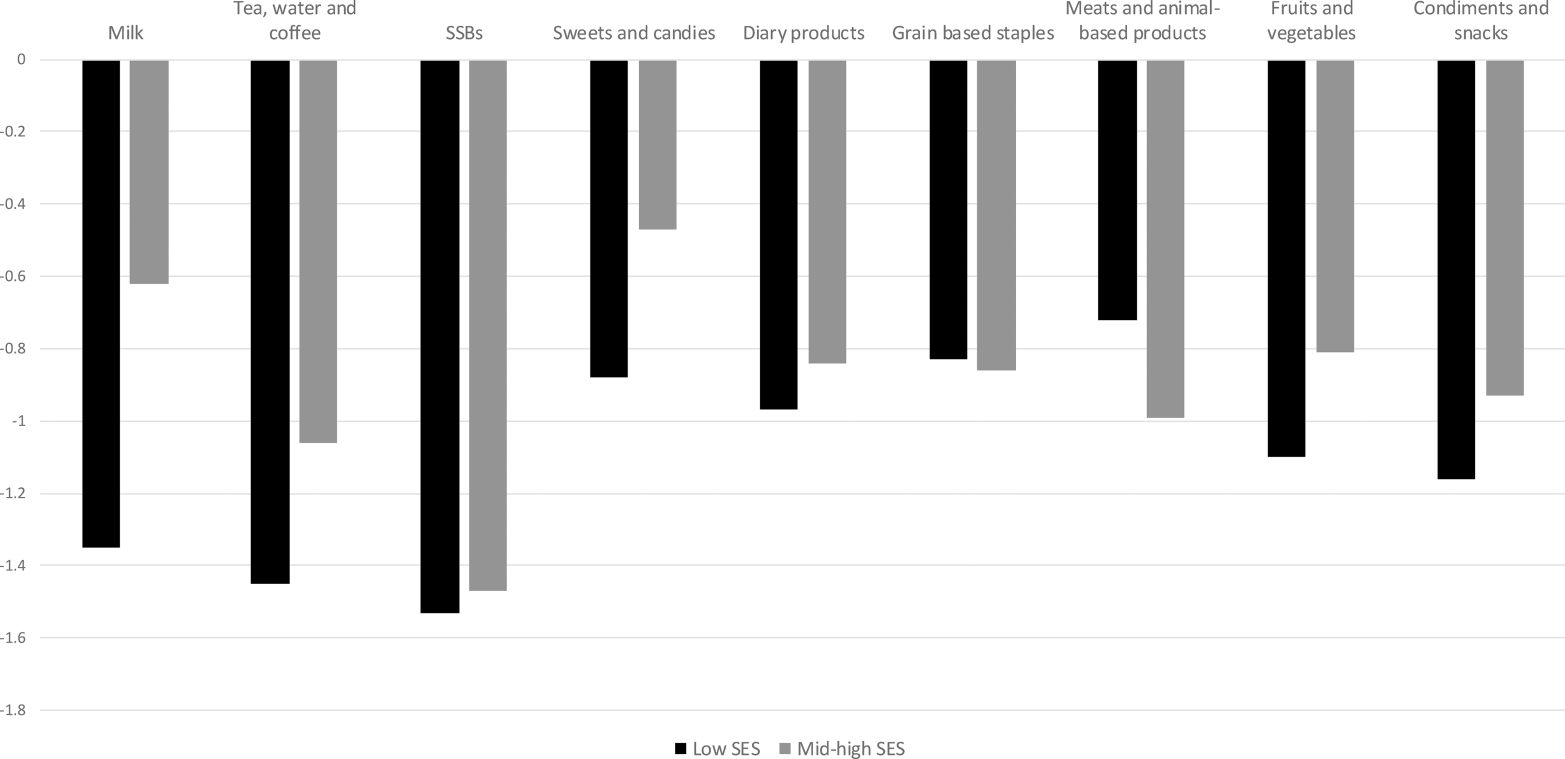
Own price elasticities by socioeconomic status. Source: Colombian Income and Expenditure Survey (ENIG) 2006–2007. SSB: sugar-sweetened beverages. p<0.1*, p<0.05**, p<0.01***.

As seen in Fig 1, low SES households have higher own-price elasticities for sweets and desserts and SSBs (as well as for fruits and vegetables, condiments and snacks, milk, tea water and coffee, and dairy products), meaning that their consumption of SSBs after a tax would reduce relatively more than in mid-high SES households (see S2 and S3 Tables for cross-price elasticities both SES groups). Furthermore, these results reflect that households from different SES have significantly different underlying preferences. One particular example is given from the fact that in mid-high SES households, SSBs and milk are complements, while the same relationship is not significant in the low SES group.

**Fig 2:**
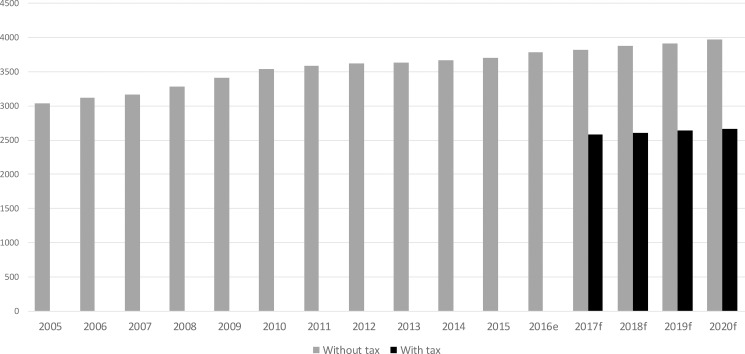
Historical and forecasted SSB volume sales per year (million liters). Source: Own estimates based on sales data from Euromonitor International. ^f^ forecasted volume.

Fig 2 shows projected SSB sales trends in the base case scenario, and our estimates considering a 20% ad-valorem tax on SSBs. Based on our results above, we expect that SSB sales would drop roughly 32% after the tax is implemented and assuming complete pass-through of the tax to prices that consumers observe, compared to the forecasted values without tax. Using the projections of both sales data and fiscal revenue, we estimate that the effect of a 20% tax on SSBs alone will yield fiscal revenues that will average 1% of Colombian government’s total revenues per year (Table 4). We can compare this result with countries that already implemented such a tax, like Chile, where a 13% ad-valorem SSB tax (effective until 2014) returned, on average, fiscal revenues of 0.47% of total revenue [53].

**Table 4:** Tax revenue simulations (tax effective 2017 onwards).

	2016	2017	2018	2019	2020
Price per liter ($ USD)	0.67	0.83	0.86	0.88	0.90
Forecasted volume (mn. lt.)	3,782	2,585 (2,884)	2,611 (2,813)	2,637 (2,942)	2,664 (2,971)
Total Fiscal revenue ($MM USD)		45,066	46,869	48,744	50,693
Revenue from SSB tax ($MM USD)		430 (479)	449 (501)	464 (518)	480 (534)

Source: Own estimates based on sales data from Euromonitor International and fiscal revenue estimates based on official data (Departamento de Impuestos y Aduanas Nacionales, 2015). Calculations based on censonsed model (uncensored model shown on parenthesis).

## Discussion

This study was designed to obtain estimates of a censored quadratic almost ideal demand system model for beverages and foods, from which price-elasticities were derived, particularly for SSBs, its substitutes and complements. We found own-price elasticities of -1.62 for SSBs, -1.05 for unsweetened unflavored milks, -1.35 for coffee and tea, -0.80 for sweets and candies (including sugar), -0.94 for dairy based products, -0.85 for grain based staples, -0.84 for animal based products, -0.96 for fruits and vegetables, and -1.01 for condiments and snacks. Our results are comparable with other studies based on a similar model specification, although to our knowledge, only a few studies estimate elasticities considering the intensive margin, namely the effect of households that might switch onto (or off) consumption, based on the prices they face. Overall, the differences between estimates are associated mostly with the modelling framework, product categorization, and data utilized [54]. In general, studies show that SSBs are price-elastic, meaning that consumptions reduces more than proportionally to price increases. Recent studies for Chile, Mexico and Ecuador report price-elasticities of -1.30, -1.16 and -1.2 respectively [30, 55, 56].

By socioeconomic level, low SES households report a higher own-price elasticity for SSBs (in absolute value) with respect to the mid-high SES group, which is consistent with SSBs being relatively more expensive lower-SES households. However, we also note that the SSBs elasticity estimate for the overall sample is greater than for any subsamples, although not significantly different, reflecting again the differences in underlying behavior amongst both groups. We observe similar patterns for sweets and candies, milk and dairy based products (which include mostly dairy based desserts, flavored milk and yogurt). The latter indicates that potentially taxing other products high in added sugar could also have a higher effect in low SES households (although the overall effect will be limited due to the fact that these goods are inelastic). In addition, we note complementarity among SSBs and sweets and candies, indicating that SSBs taxation will have additional health benefits by reducing consumption of added sugar in other foods (although it will also will reduce milk consumption). Also, there is some substitution between SSBs and other food groups, implying that households will reallocate consumption from SSBs to other foods like grains, fruits and vegetables, and condiments and snacks.

Our study shows that a 20% SSB tax would reduce significantly their consumption (roughly 1,197 million liters), and therefore may meaningfully lower the prevalence of obesity and related non-communicable diseases, and reducing associated healthcare costs. The tax could also provide the Colombian government with an additional 1% of total revenues per year on average. Moreover, there are also additional health benefits beyond the direct effect of reduction in SSB consumption (by reducing the consumption of other sugary foods, for example), which can be further boosted if the Colombian government directs these tax revenues towards investments in national and local health care systems. Future initiatives could further include other foods in the tax base, such as sweets and candies. Recent evidence indicates that such strategy is more effective to reduce added sugar consumption, than a large tax on SSBs alone [56]. In terms of policy practice, taxes on SSBs can eventually have enough support to be implemented, while a tax on other industries (candies, sweets) seems more complex in the short and medium run. This paper contributes as a first step in a strategy to promote healthier behaviors in the Colombian population.

This work has some limitations. First, the data does not allow us to further separate food items that have added sugar from artificially sweetened products (e.g., we cannot separate regular sodas from diet sodas), even though diet soda consumption in Colombia represent around 13–15% of total SSB consumption [33]. Second, the data is from 2006–07 and so there may be sufficient changes in the economic development and preferences of consumers in the past decade. However, growth in Colombia has been slow, ranging between -0.8% (minimum) to 2.7% (maximum) in the past decade, in part due to the internal armed conflict that only recently achieved a peace deal. Even with the peace deal, there is much recovery that reinvestments that the country will need to make before significant growth will occur. As such, we do not think that consumer demand would have changed dramatically and that these estimates are useful for using to forecast how a SSB tax might change purchases and generate revenue, particularly in SSBs penetration increases significantly in the following years, as it has happened in similar middle-income countries [23].

Third, our model imposed standard economic demand theory (adding-up, symmetry and homogeneity restrictions) although it is not necessarily expected for these constraints to be satisfied empirically for a particular data source. However, recent literature shows that the rejection of these restrictions is often due to model specification rather that the true nature of data [57, 58]. In the case of adding-up, [59] note that while this restriction is unlikely to hold, it only represents an estimation difficulty if the system is estimated excluding one equation, which is not out case.

Finally, is important to consider that our model does not account for other types of household and industry behavior. In particular, individuals or households could switch between different product varieties with significantly different nutrient composition, moving to items with lower price and lesser nutritional value. In particular, we assumed that the tax is passed fully to final prices, although recent evidence indicates that pass-through is often imperfect [60]. Also, we cannot in any way examine leakages in the system (e.g., black marketing of selected products or bringing across the border untaxed items illegally). However, unlike cigarettes, food and beverage items are fairly bulky, and we do not expect excessive smuggling or other similar leakages.

Globally, the shift towards highly processed diets with excessive sodium, added sugar, unhealthy fats and highly refined carbohydrates has defined the global dietary shift of the past several decades [61–63]. A new paradigm involving healthier diets requires a large number of changes, including ultimately shifts in the culture of eating, a reduction of marketing of unhealthy foods and beverages, front-of-package labeling of either healthy or unhealthy foods and beverages, and shifts in the relative prices to encourage significant changes toward healthier food purchases [22]. The design of a meaningful SSBs tax to enhance the overall effect into a healthier Colombian diet is an important step forward This study has shown that a 20% tax on SSBs should prove to be effective, and yield a non-negligible revenue (about 1% of total fiscal revenue per year, based on volume sales data) that could be potentially used towards public health promotion and investments. Recently, a tax reform package has been introduced in Colombia, which includes approximately an 18% tax on SSBs, which was debated in the congress but rejected in early-2017. There may be future efforts to reconsider this, and if such a tax is implemented, rigorous careful evaluation is needed to understand if it is able to lower the consumption of unhealthy sugary beverages.

## Supporting information

S1 Table Uncompensated elasticities from QUAIDS uncensored model. (PDF)

S2 Table Uncompensated elasticities from QUAIDS censored model (low SES group). (PDF)

S3 Table Uncompensated elasticities from QUAIDS censored model (mid-high SES group). (PDF)
