# Supplementary material for: Sugary drinks taxation, projected consumption and fiscal revenues in Colombia: Evidence from a QUAIDS model
Source: PLoS One. 2017 Dec 20;12(12):e0189026. doi: 10.1371/journal.pone.0189026 (PMC5737888; doi:10.1371/journal.pone.0189026)
Supplement: S1 Table — (PDF) [file pone.0189026.s001.pdf]

S1 Table. Uncompensated elasticities from QUAIDS uncensored model

	Change in price								
Change in quantity	**Milk**	**Tea and coffee**	**SSBs**	**Sweets and candies**	**Diary-based products**	**Grain based staples**	**Meat and animal products**	**Fruits and vegetables**	**Condiments and snacks**
**Milk**	**-1.205*** **	0.112	-0.129	-0.337***	-0.115	0.37**	1.764***	1.413***	0.078
	**0.16**	0.096	0.109	0.076	0.117	0.151	0.302	0.275	0.097
**Tea, water and coffee**	0.222	**-1.184*** **	0.347**	0.17	-0.038	-0.251	-1.818**	-1.771**	-0.17
	0.293	**0.256**	0.173	0.199	0.182	0.226	0.831	0.908	0.148
**SSBs**	0.766	-0.149	**-1.225*** **	0.772***	0.232	-0.131	-3.377***	-3.865***	-0.161
	0.509	0.285	**0.367**	0.297	0.33	0.404	1.125	1.253	0.245
**Sweets and candies**	-0.139	-0.05	-0.093	**-0.606*** **	0.074	0.001	0.614***	1.046***	-0.046
	0.097	0.053	0.064	**0.081**	0.066	0.086	0.189	0.194	0.052
**Diary-based products**	-0.238***	0.071	-0.009	-0.138***	**-0.928*** **	0.079	0.636***	0.74***	0.032
	0.076	0.044	0.052	0.041	**0.056**	0.071	0.176	0.163	0.043
**Grain based staples**	-0.05	0.062	-0.006	-0.172***	-0.001	**-0.814*** **	0.367	0.616***	0.044
	0.072	0.045	0.045	0.049	0.05	**0.05**	0.225	0.208	0.04
**Meat and animal products**	0.117***	-0.024	0.018	0.003	-0.021	-0.165***	**-1.025*** **	-0.404***	-0.019
	0.039	0.021	0.021	0.033	0.022	0.031	**0.167**	0.146	0.016
**Fruits and vegetables**	-0.134***	0.031	-0.006	0.028	-0.01	-0.036	-0.293**	**-0.884*** **	0.005
	0.042	0.019	0.022	0.03	0.025	0.028	0.132	**0.15**	0.014
**Condiments and snacks**	0.834**	-0.45*	0.242	0.721***	0.008	-0.324	-3.44***	-3.709***	**-1.101*** **
	0.374	0.238	0.255	0.201	0.287	0.357	0.754	0.792	**0.27**

Source: Colombian Income and Expenditure Survey (ENIG) 2006-2007. Note: SSB: Sugar-sweetened beverages; bold denote own-price elasticities; p<0.1*, p<0.05**, p<0.01***. Total sample: 33,824 households.
