# Supplementary material for: Sugary drinks taxation, projected consumption and fiscal revenues in Colombia: Evidence from a QUAIDS model
Source: PLoS One. 2017 Dec 20;12(12):e0189026. doi: 10.1371/journal.pone.0189026 (PMC5737888; doi:10.1371/journal.pone.0189026)
Supplement: S2 Table — (PDF) [file pone.0189026.s002.pdf]

S2 Table. Uncompensated elasticities from QUAIDS censored model (low SES group)

Change in quantity	Change in price								
	**Milk**	**Tea and coffee**	**SSBs**	**Sweets and candies**	**Diary-based products**	**Grain based staples**	**Meat and animal products**	**Fruits and vegetables**	**Condiments and snacks**
**Milk**	**-1.353*****	0.107*	-0.113	-0.022	0.068	0.409***	1.141***	0.60***	0.091
	**0.163**	0.06	0.073	0.062	0.075	0.093	0.266	0.22	0.059
**Tea, water and coffee**	0.451	**-1.45*****	0.021	0.078	-0.01	-0.275	-0.933	-0.432	-0.111
	0.36	**0.194**	0.149	0.129	0.129	0.21	0.692	0.585	0.099
**SSBs**	0.232	-0.014	**-1.531*****	-0.171*	0.331***	0.309***	-0.396	-0.069	0.101**
	0.171	0.061	**0.123**	0.091	0.082	0.103	0.315	0.262	0.045
**Sweets and candies**	-0.124	-0.027	-0.242***	**-0.885*****	0.181***	0.07	0.41**	0.716***	-0.06
	0.113	0.047	0.056	**0.079**	0.058	0.066	0.189	0.161	0.04
**Diary-based products**	-0.181**	0.06*	0.095***	-0.027	**-0.971*****	0.052	0.326**	0.261**	0.008
	0.086	0.033	0.033	0.035	**0.045**	0.046	0.151	0.124	0.025
**Grain based staples**	-0.105	0.052*	0.021	-0.067***	0.009	**-0.829*****	0.134**	0.325**	0.044
	0.067	0.03	0.035	0.025	0.038	**0.041**	0.115	0.104	0.026
**Meat and animal products**	0.013	0.044***	-0.038*	-0.079***	0.001	-0.122***	**-0.721*****	-0.09*	0.005
	0.035	0.015	0.02	0.024	0.023	0.023	**0.065**	0.053	0.011
**Fruits and vegetables**	-0.053	0.04	0.054*	0.041	-0.021	-0.037	-0.396**	**-1.097*****	-0.004
	0.095	0.031	0.032	0.032	0.031	0.04	0.17	**0.161**	0.019
**Condiments and snacks**	1.464***	-0.432**	0.588***	0.31*	-0.336	-0.496*	-2.794***	-2.405***	**-1.16*****
	0.448	0.194	0.226	0.162	0.245	0.284	0.755	0.693	**0.245**

Source: Colombian Income and Expenditure Survey (ENIG) 2006-2007. Note: SSB: Sugar-sweetened beverages; bold denote own-price elasticities; p<0.1*, p<0.05**, p<0.01***. Sub-sample: 20,562 households.
