# Supplementary material for: Sugary drinks taxation, projected consumption and fiscal revenues in Colombia: Evidence from a QUAIDS model
Source: PLoS One. 2017 Dec 20;12(12):e0189026. doi: 10.1371/journal.pone.0189026 (PMC5737888; doi:10.1371/journal.pone.0189026)
Supplement: S3 Table — (PDF) [file pone.0189026.s003.pdf]

S3 Table. Uncompensated elasticities from QUAIDS censored model (mid-high SES group)

	Change in price								
Change in quantity	**Milk**	**Tea and coffee**	**SSBs**	**Sweets and candies**	**Diary-based products**	**Grain based staples**	**Meat and animal products**	**Fruits and vegetables**	**Condiments and snacks**
**Milk**	**-0.626*****	0.055	-0.404***	-0.123*	-0.014	0.295***	1.085***	0.639***	-0.024
	**0.144**	0.122	0.141	0.071	0.119	0.107	0.255	0.196	0.067
**Tea, water and coffee**	-0.028	**-1.064*****	0.411	-0.014	0.082	-0.105	-0.536	-0.339	-0.03
	0.154	**0.271**	0.25	0.13	0.213	0.213	0.73	0.662	0.092
**SSBs**	-0.305	0.182	**-1.47*****	-0.193	0.295*	0.228	-0.539	-0.09	0.162**
	0.202	0.122	**0.234**	0.137	0.16	0.141	0.372	0.364	0.073
**Sweets and candies**	-0.233*	-0.141	-0.279**	**-0.471*****	0.311***	0.014	0.665***	0.917***	-0.088
	0.133	0.104	0.133	**0.139**	0.109	0.104	0.202	0.18	0.064
**Diary-based products**	-0.235***	0.034	0.047	-0.082*	**-0.846*****	0.072	0.283	0.299*	-0.008
	0.07	0.065	0.079	0.044	**0.065**	0.064	0.179	0.154	0.032
**Grain based staples**	0.063	0.043	0.09	-0.097**	-0.063	**-0.866*****	-0.034	0.057	0.016
	0.06	0.049	0.084	0.047	0.068	**0.078**	0.246	0.223	0.025
**Meat and animal products**	0.082*	-0.007	0.014	0.036	-0.094*	-0.116**	**-0.997*****	-0.352*	0.004
	0.042	0.041	0.061	0.034	0.052	0.051	**0.221**	0.168	0.022
**Fruits and vegetables**	-0.138***	0.016	0.11**	-0.006	-0.022	-0.068*	-0.309**	**-0.814*****	0.01
	0.052	0.028	0.053	0.038	0.046	0.041	0.132	**0.113**	0.021
**Condiments and snacks**	0.095	-0.16	0.479**	0.255**	-0.213	-0.283	-1.302***	-1.136**	**-0.929*****
	0.18	0.167	0.217	0.106	0.201	0.192	0.434	0.472	**0.103**

Source: Colombian Income and Expenditure Survey (ENIG) 2006-2007. Note: SSB: Sugar-sweetened beverages; bold denote own-price elasticities; p<0.1*, p<0.05**, p<0.01***. Sub-sample: 13,262 households.
